# Neural Mobilization for Reducing Pain and Disability in Patients with Lumbar Radiculopathy: A Systematic Review and Meta-Analysis

**DOI:** 10.3390/life13122255

**Published:** 2023-11-26

**Authors:** Long-Huei Lin, Ting-Yu Lin, Ke-Vin Chang, Wei-Ting Wu, Levent Özçakar

**Affiliations:** 1 Kaohsiung Rukang Physiotherapy Clinic, Kaohsiung 83050, Taiwan; cosx9954022@gmail.com; 2 Department of Physical Medicine and Rehabilitation, Lotung Poh-Ai Hospital, Yilan 26546, Taiwan; t840326@icloud.com; 3 Department of Physical Medicine and Rehabilitation, National Taiwan University Hospital, College of Medicine, National Taiwan University, Taipei 10048, Taiwan; wwtaustin@yahoo.com.tw; 4 Department of Physical Medicine and Rehabilitation, National Taiwan University Hospital, Bei-Hu Branch, Taipei 10845, Taiwan; 5 Center for Regional Anesthesia and Pain Medicine, Wang-Fang Hospital, Taipei Medical University, Taipei 11696, Taiwan; 6 Department of Physical and Rehabilitation Medicine, Hacettepe University Medical School, Ankara 06100, Turkey; lozcakar@yahoo.com

**Keywords:** sciatica, radiculopathy, manual therapy, physical therapy, peripheral nerve injury

## Abstract

Lumbar radiculopathy causes lower back and lower extremity pain that may be managed with neural mobilization (NM) techniques. This meta-analysis aims to evaluate the effectiveness of NM in alleviating pain and reducing disability in patients with lumbar radiculopathy. We hypothesized that NM would reduce pain and improve disability in the lumbar radiculopathy population, leveraging the statistical power of multiple studies. Electronic databases from their inception up to October 2023 were searched for randomized controlled trials (RCTs) that explored the impact of NM on lumbar radiculopathy. Our primary outcome measure was the alteration in pain intensity, while the secondary one was the improvement of disability, standardized using Hedges’ *g*. To combine the data, we employed a random-effects model. A total of 20 RCTs comprising 877 participants were included. NM yielded a significant reduction in pain intensity (Hedges’ *g* = −1.097, 95% CI = −1.482 to −0.712, *p* < 0.001, $I^{2\;}$= 85.338%). Subgroup analyses indicated that NM effectively reduced pain, whether employed alone or in conjunction with other treatments. Furthermore, NM significantly alleviated disability, with a notable effect size (Hedges’ *g* = −0.964, 95% CI = −1.475 to −0.453, *p* < 0.001, $I^{2}$ = 88.550%), particularly in chronic cases. The findings provide valuable insights for clinicians seeking evidence-based interventions for this patient population. This study has limitations, including heterogeneity, potential publication bias, varied causal factors in lumbar radiculopathy, overall study quality, and the inability to explore the impact of neural pathology on NM treatment effectiveness, suggesting opportunities for future research improvements.

## 1. Introduction

Lumbar radiculopathy is a debilitating condition characterized by the compression of lumbar nerve roots [1]. Causes of lumbar radiculopathy encompass bulging disc, herniation, hypertrophic facet or adjacent ligaments, spondylolisthesis, and, in rare instances, neoplastic and infectious conditions [1]. Lumbar radiculopathy is often referred to as sciatica, a term derived from its hallmark symptom [2]. It manifests as radiating leg pain over the distribution of the sciatic nerve [3]. Treatment options for lumbar radiculopathy encompass conservative approaches, pharmaceutical interventions, and surgical procedures. Conservative care options are diverse, encompassing various exercise protocols, the use of electrical modalities like transcutaneous electrical nerve stimulation, and techniques focused on mobilizing the affected tissue, including spinal mobilization and neural mobilization (NM) [4].

NM is a manual therapy technique designed to simultaneously lengthen a nerve at one joint while shortening it at an adjacent joint, with the primary objective of enhancing the smooth movement of neural structures within the surrounding tissues [5]. This approach typically comprises two techniques, i.e., “slider” and “tensioner” [6]. The former focuses on facilitating the sliding motion of neural tissue in relation to neighboring structures without generating significant tension [7]. Conversely, the latter technique aims to create neural tension by increasing the distance between each end of the nerve tract, always within the tissue’s elastic limits, ultimately enhancing the nerve’s viscoelastic properties [7]. The mechanisms underlying NM interventions involve restoring the equilibrium between the nerve and nearby connective tissues and reducing intraneural edema by dispersing fluid within the nerve axon [8]. Furthermore, studies have indicated that NM has a hypoalgesic effect and can reduce the mechanical sensitivity of nerves [9,10]. While there have previously been systematic reviews and meta-analyses addressing related topics [11,12], none have specifically focused on the lumbar radiculopathy population, nor have they delved into the further exploration of NM types, regimens, and lumbar radiculopathy symptom stages. Therefore, the primary aim of our meta-analysis is to investigate the benefits of NM on pain intensity and disability in individuals with lumbar radiculopathy. Furthermore, we aim to examine whether there are differential benefits based on NM type, regimen, and lumbar radiculopathy symptom stage.

## 2. Methods

### 2.1. Search Strategy

We performed an extensive literature review in accordance with the 2020 Preferred Reporting Items for Systematic Reviews and Meta-Analyses (PRISMA) guidelines, spanning from the inception of the databases to October 2023 [13]. The PRISMA checklist is provided in Table S1. Our research protocol was duly registered on Inplasy.com with the registration number INPLASY2023100039. The screening process involved two reviewers, L.-H.L. and T.-Y.L., who meticulously examined various databases, including PubMed, ClinicalTrials.gov, Cochrane Library, and the Physiotherapy Evidence Database (PEDro). Our search strategy incorporated the following keywords: (“neural mobilization techniques” OR “neurodynamic mobilization techniques” OR “nerve mobilization techniques”) AND (“lumbar radiculopathy” OR “sciatica”). To ensure a comprehensive search, we employed different variations of these terms. Detailed information regarding the literature search is given in Table S2.

### 2.2. Inclusion and Exclusion Criteria

The PICO (population, intervention, comparison, outcome) setting of the current meta-analysis was as follows: P: human participants with lumbar radiculopathy; I: the NM technique; C: controls that did not employ NM; and O: changes in pain intensity and disability.

The inclusion criteria were as follows: (1) RCTs investigating pain intensity and disability before/after NM; (2) enrolling adults diagnosed with lumbar radiculopathy and/or sciatica based on radiography, reproducing radiated symptoms in the leg with a passive straight leg raise test or slump test; (3) intervention groups being treated with NM alone or NM plus other treatments; (4) at least one reference group using treatments other than NM (inclusion of both passive control, such as no intervention, and active control, such as conventional treatments).

The exclusion criteria were as follows: (1) non-RCTs; (2) studies that enrolled patients with recent associated neurological symptoms (e.g., foot drop and cauda equina), previous surgery in the lumbar spine, spine fractures, and lower extremity injuries that induced leg pain; (3) case reports, case series, and trials using quasi-experimental, single-arm, or longitudinal follow-up designs; (4) studies lacking the desired outcome; and (5) those that enrolled participants duplicated with a previously published trial.

### 2.3. Primary Outcome Measurements

The primary focus of the study centered on evaluating alterations in pain intensity, quantified using the Numeric Rating Scale (NRS) and the Visual Analog Scale (VAS) before and after the intervention [14]. Detailed descriptions can be found in Table S3 for clarity.

### 2.4. Secondary Outcome Measurements

The secondary outcome involved assessing changes in disability, measured by the Oswestry Disability Index (ODI) [15], Modified Oswestry Disability Index (MODI) [16], Quebec Back Pain Disability Scale (QBPDS) [17], Roload Morris Disability Questionnaire (RMDQ) [18], 36-Item Short Form Survey (SF-36) [19], and 12-Item Short Form Survey (SF-12). Elaborate explanations are systematically arranged in Table S3 to enhance clarity.

### 2.5. Data Extraction

The data extraction process was conducted by two independent reviewers (L.-H.L. and T.-Y.L.). This process involved gathering information encompassing demographics, study design, intervention details, outcome measures, and assessment timeframes. We utilized Excel to create the data collection form. In cases of disagreement between the two reviewers, consensus was achieved through discussion or consultation with the corresponding author for resolution. For studies with multiple arms, we organized similar eligible sets to facilitate straightforward pairwise comparisons, as previously reported [20]. In situations where we encountered missing data in the published articles, we reached out their corresponding authors to obtain the original data. If the need arose to convert medians and interquartile ranges into means and standard deviations, we followed the guidelines provided in the Cochrane Handbook for Systematic Reviews of Interventions [21].

### 2.6. Assessment and Quality Classification

The evaluation of the quality of the Randomized Controlled Trials (RCTs) included was performed using the Cochrane risk-of-bias tool, known as RoB 2. RoB 2 assesses six primary criteria, namely the randomization process, intervention adherence, missing outcome data, outcome measurement, selective reporting, and the overall risk of bias [22]. In the assessment of intervention adherence within the RoB 2 tool, two options were available: the intention-to-treat approach, which is based on intervention assignment, and the per-protocol approach, which is based on intervention adherence. For our meta-analysis, we used the per-protocol approach, which was in alignment with the study design used in the majority of the included studies [22].

### 2.7. Statistical Analysis

Due to the diverse measurement tools in the included studies, we deemed the mean difference inappropriate and opted for Hedges’ g as our standardized effect size. This choice ensures outcome comparability across studies with varied measurement scales. Our results, expressed with Hedges’ g and corresponding 95% CIs, quantify and convey the study findings. Effect sizes of 0.2, 0.5, and 0.8 were construed as representative of small, moderate, and large effects, respectively [23]. This study employed a random-effects model. This choice was based on the assumption that there was a distribution of intervention effects across studies, accommodating variability. The model recognizes that observed differences stem from both chance and genuine variation in intervention effects, resulting in broader confidence intervals when heterogeneity is present, as opposed to a fixed-effect model [24]. Additionally, we assessed the extent of heterogeneity among the studies by employing I^2^ and Cochran’s Q statistics, with I^2^ values exceeding 50% signifying significant heterogeneity [25]. To appraise the influence of individual trials on the overall effect size, we conducted sensitivity analyses using the one-study removal method. Subgroup analyses were also carried out based on the type of NM techniques (slider or tensioner, slump or straight leg raise), symptom stage (chronic stage > 3 months, non-chronic stage ≤ 3 months), and NM regimen (NM only and NM plus). Furthermore, we performed meta-regression analyses to investigate potential associations between the pain-alleviating effects of NM and the duration of treatment per day, as well as the frequency of sessions per week. In order to scrutinize the potential presence of publication bias, we visually assessed the funnel plots and examined the statistics derived from Egger’s regression test [26]. Statistical analysis was performed using Comprehensive Meta-Analysis software (version 3, Biostat, Englewood, NJ, USA), with statistical significance defined as a two-tailed *p*-value below 0.05.

## 3. Results

### 3.1. Study Identification and Selection

**Figure 1:**
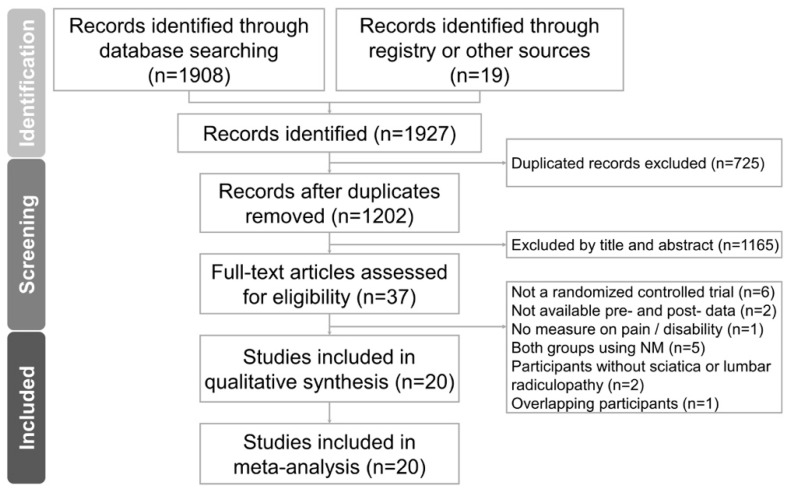
PRISMA flow diagram describing the screening and review process for the current meta-analysis.

Figure 1 displays the PRISMA flowchart illustrating our literature search. Initially, we identified 1202 non-duplicated citations through our research efforts. Subsequently, we subjected 37 articles to further scrutiny for eligibility. After conducting a thorough full-text assessment, we excluded seventeen articles for various reasons: six were not RCTs, two did not provide both pre- and post-intervention data, one did not report the desired outcome measurements, five lacked a non-NM control group, two did not include patients with lumbar radiculopathy, and one had participants who overlapped with another publication by the same author. The specific rationales for these exclusions are given in Table S4. We ultimately incorporated 20 RCTs into our analysis, involving a total of 877 participants whose ages ranged from 20 to 60 years. The intervention durations spanned from two to six weeks. Among the trials, one was a three-arm study, while the remaining nineteen were two-arm RCTs.

The aforementioned three-arm study compared three interventions: NM slider technique, NM tensioner technique, and stretch exercises [27]. Within the group of two-arm studies, six compared NM to conventional treatments, which included physical agents combined with lumbopelvic region exercises such as lumbopelvic muscle strengthening, stretching, or range-of-motion exercises [28,29,30,31,32,33]. Additionally, one study compared NM to physical agents (e.g., transcutaneous electrical nerve stimulation) along with lumbar region massage [34]; another study examined NM versus lumbar range-of-motion exercises combined with lumbar stabilization exercises [35]; one study involved education about daily-life activities as the control [36]; and one study combined conventional treatments with lumbar region trigger point release and hamstring stretching [37]. Similarly, another study paired conventional treatments with hamstring stretching [38], one study explored NM versus kinesio taping [39], three studies focused on NM versus lumbar stabilization exercises [40,41,42], three studies compared NM to physical agents [43,44,45], and one study combined conventional treatments with massage [46].

Regarding pain intensity measurement, nine studies used the NRS [28,32,33,34,38,41,42,45,46], whereas ten studies used the VAS [27,29,30,31,35,36,37,39,43]. Regarding disability measurement, six studies used the ODI [27,29,31,36,37,38], three studies used the MODI [32,33,43], one study used the SF-36 [40], one study used the SF-12 [28], one study used the QBPDS [45], and two studies used the RMDQ [41,42]. The characteristics of the included studies are listed in Table 1 and Table 2.

### 3.2. Methodological Quality of the Included Studies

With respect to the overall methodological quality of the included studies, we found that 15% had a low risk of bias (n = 3), 20% (n = 4) had some risk of bias, and 65% had a high risk of bias (n = 13) (Figure S1). The item that was mostly rated as some risk of bias was missing outcome data, followed by an absence of explanation for baseline differences and a lack of clear description of allocation concealment. The item that was mostly rated as high risk of bias was no information about the extent of missing outcome data. The details of the risk of bias assessment are summarized in Table 3.

### 3.3. Effectiveness of NM on Pain Reduction

**Figure 2:**
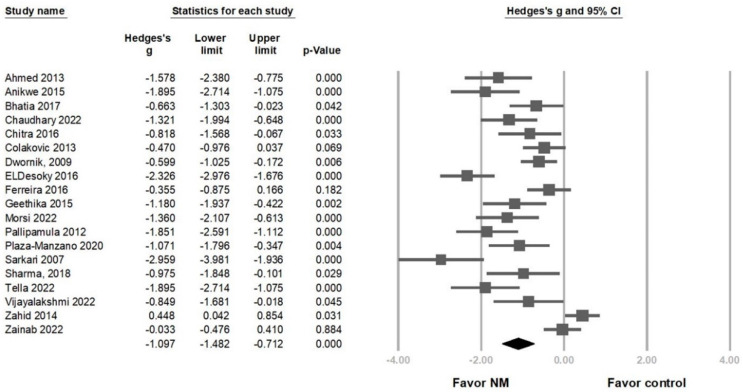
Forest plot of the overall effects of neural mobilization (NM) on pain reduction in patients with lumbar radiculopathy [27,28,29,30,31,32,33,34,35,36,37,38,39,41,42,43,44,45,46].

**Figure 3:**
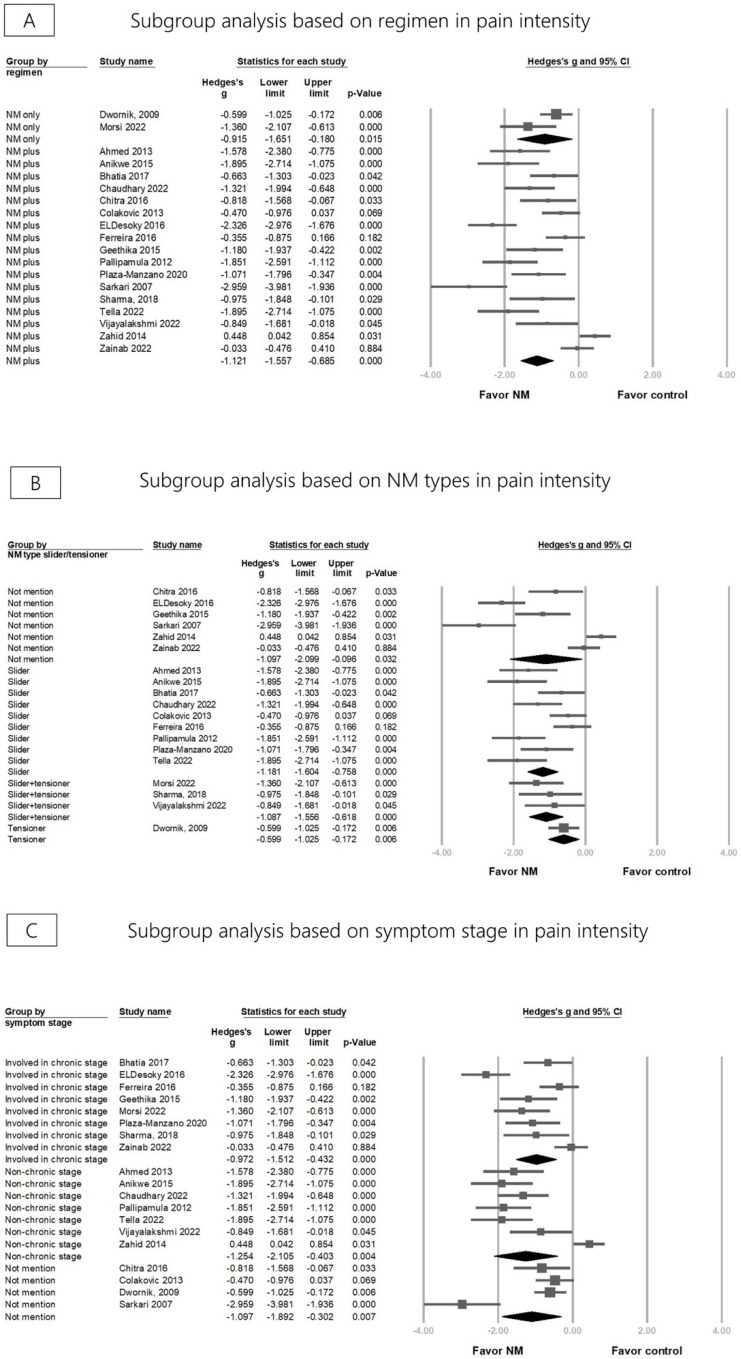
Forest plot of subgroup analysis for pain intensity based on the type/regimen of neural mobilization (NM) and symptom stage of lumbar radiculopathy [27,28,29,30,31,32,33,34,35,36,37,38,39,41,42,43,44,45,46].

Overall, pain intensity was significantly reduced in the NM group in 19 RCTs (Hedges’ *g* = −1.097, 95% CI = −1.482 to −0.712, *p* < 0.001, $I^{2}\;$= 85.338%) (Figure 2). A sensitivity analysis was conducted using the one-study removal method and showed a consistently significant effect of NM on pain reduction (Figure S2). Subgroup analyses considered regimen, NM technique types, and symptom stages. Both groups using NM alone and NM in combination with other treatments (e.g., physical agents or lumbar stabilization exercises) showed a significant pain reduction (Hedges’ *g* = −0.915, 95% CI = −1.651 to −0.180, *p* = 0.015, *I*^2^ = 66.808%; Hedges’ *g* = −1.121, 95% CI = −1.557 to −0.685, *p* < 0.001, *I*^2^ = 86.639%) (Figure 3A). The effect sizes for pain reduction were −1.181 (95% CI = −1.604 to −0.758, *p* < 0.001, *I*^2^ = 71.659%) for the groups using the slider technique exclusively, −1.087 (95% CI = −1.556 to −0.618, *p* < 0.001, *I*^2^ < 0.001%) for the groups employing the slider and tensor techniques in combination, −0.599 (95% CI = −1.025 to −0.172, *p* = 0.006, *I*^2^ < 0.001%) for the group using the tensioner technique exclusively, and −1.097 (95% CI = −2.099 to −0.096, *p* = 0.032, *I*^2^ = 93.864%) for the group where the therapeutic technique was not specifically mentioned (Figure 3B). Regarding pain reduction in different stages, the effect size of the chronic stage group was −0.972 (95% CI = −1.512 to −0.432, *p* < 0.001, *I*^2^ = 82.279%) and that of the non-chronic stage group was −1.254 (95% CI = −2.105 to −0.403, *p* = 0.004, *I*^2^ = 90.573%). The effect size for the group that did not specifically mention the stage was −1.097 (95% CI = −1.892 to −0.302, *p* = 0.007, *I*^2^ = 84.685%) (Figure 3C). A meta-regression analysis was conducted to investigate whether the duration of treatment (16 RCTs) and frequency of NM sessions per week (13 RCTs) could modify the effects of pain reduction. The regression coefficient was −0.049 (95% CI = −0.065 to –0.033, *p* < 0.001) for treatment duration in days and −0.354 (95% CI = −0.488 to −0.219, *p* < 0.001) for session frequency (per week), indicating that increased NM intervention duration and sessions per week contributed to greater pain reduction (Figures S3 and S4).

### 3.4. Effectiveness of NM on Disability

**Figure 4:**
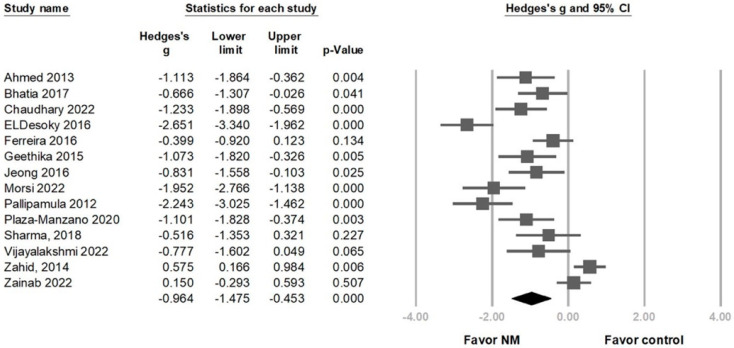
Forest plot of the overall effects of neural mobilization (NM) on relief of disability in patients with lumbar radiculopathy [27,28,29,31,32,33,36,37,38,40,41,42,43,45].

**Figure 5:**
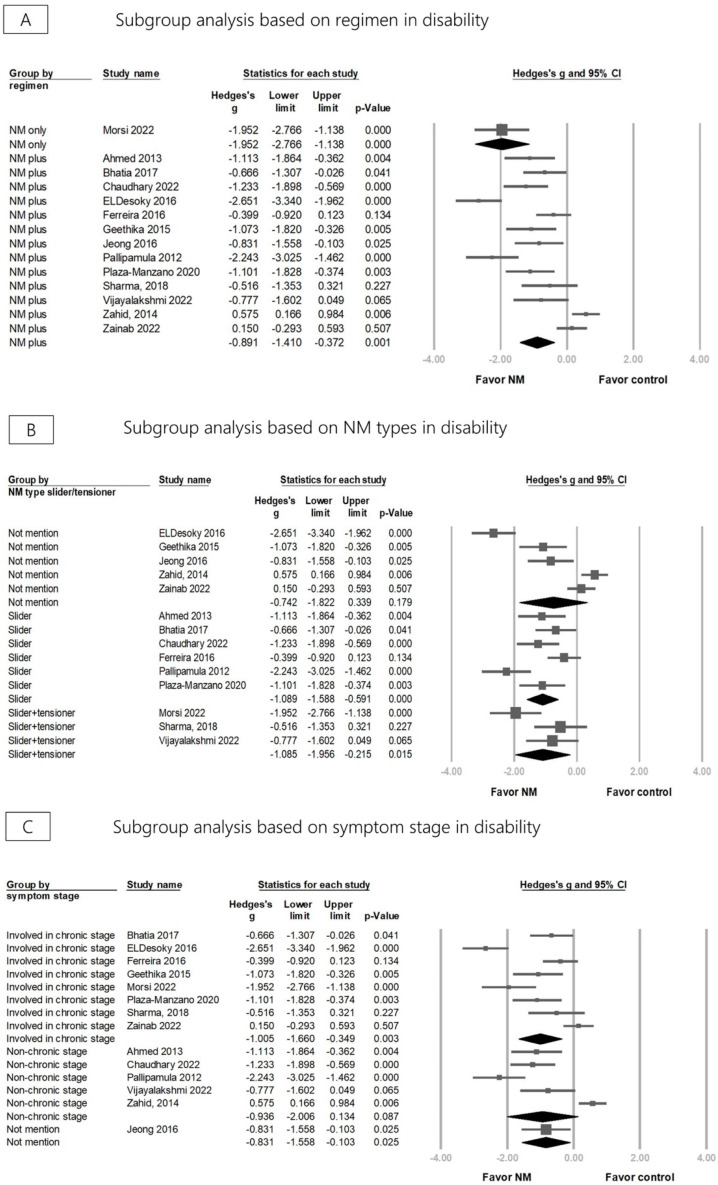
Forest plot of subgroup analysis for disability based on the type/regimen of neural mobilization (NM) and symptom stage of lumbar radiculopathy [27,28,29,31,32,33,36,37,38,40,41,42,43,45].

Compared with the control group, disability was significantly reduced after NM in 14 RCTs (Hedges’ *g* = −0.964, 95% CI = −1.475 to −0.453, *p* < 0.001, $I^{2}\;$= 88.550%) (Figure 4). Sensitivity analysis using the one-study removal method consistently confirmed a significant effect of NM on disability (Figure S5). Subgroup analysis divided by different regimens revealed that using NM alone and NM combined with other treatments showed a significant disability improvement (Hedges’ *g* = −1.952, 95% CI = −2.766 to −1.138, *p* < 0.001, *I*^2^ < 0.001%; Hedges’ *g* = −0.891, 95% CI = −1.410 to −0.372, *p* = 0.001, *I*^2^ = 88.410%) (Figure 5A). The effect sizes for disability improvement were −1.089 (95% CI = −1.588 to −0.591, *p* < 0.001, *I*^2^ = 69.707%) for the group using the slider technique exclusively, −1.085 (95% CI = −1.956 to −0.215, *p* = 0.015, *I*^2^ = 70.014%) for the group employing both techniques in combination, and −0.742 (95% CI = −1.822 to 0.339, *p* = 0.179, *I*^2^ = 94.476%) for the group where the therapeutic technique was not specifically mentioned (Figure 5B). The effect sizes for disability improvement were −1.005 (95% CI = −1.660 to −0.349, *p* = 0.003, *I*^2^ = 87.745%) for the chronic stage group, −0.936 (95% CI = −2.006 to 0.134, *p* = 0.087, *I*^2^ = 92.485%) for the non-chronic stage group, and −0.831 (95% CI = −1.558 to −0.103, *p* = 0.025, *I*^2^ < 0.001%) for the group where the stage was not specifically mentioned (Figure 5C).

Regarding the result of meta-regression, the regression coefficient was −0.043 (95% CI = −0.062 to −0.025, *p* < 0.001) for treatment duration in days (12 RCTs) and −0.331 (95% CI = −0.506 to −0.156, *p* < 0.001) (10 RCTs) for session frequency (per week), indicating that increased NM intervention duration and sessions per week contributed to a greater disability improvement (Figures S6 and S7).

### 3.5. Publication Bias

The funnel plot for pain intensity and disability revealed an asymmetry of effect size distribution, with both *p*-values < 0.001 according to Egger’s regression test (Figures S8 and S9).

## 4. Discussion

Our meta-analysis revealed that the use of NM significantly reduced both pain and disability in lumbar radiculopathy. Regardless of the specific regimen and the type of technique employed, NM consistently produced positive results. Longer treatment duration and more frequent sessions further enhanced the effectiveness of NM in reducing pain and improving disability. Regarding disability, while the effect size of NM for participants in the chronic stage group was significant, it lacked significance for participants in the non-chronic stage group. Moreover, the concept of clinical significance is closely intertwined with effect size estimation, which, in turn, leads to power analysis estimation. Generally, effect sizes of 0.2, 0.5, and 0.8 are considered indicative of small, moderate, and large effects, respectively [23]. In the current meta-analysis, the effect size of pain intensity reduction (Hedges’ *g* = −1.097) and disability improvement (Hedges’ *g* = −0.964) was large. This suggests their clinical significance. To the best of our knowledge, this article represents the first meta-analysis investigating the effectiveness of NM in reducing pain and disability specifically in individuals with lumbar radiculopathy. Moreover, this study includes subgroup analyses, addressing a significant gap in the current literature.

In a previous systematic review and meta-analysis, Neto et al. [11] included 10 RCTs wherein the effects of NM targeting the lower body quadrant in both healthy individuals and those with low back pain were examined. The results indicated that NM had moderate effects on flexibility in healthy adults. Notably, it led to a significant pain reduction and disability improvement in individuals with low back pain, highlighting the potential benefits of NM. Pourahmadi et al. [12] conducted a systematic review and meta-analysis of seven RCTs. Examining the effectiveness of NM for patients with low back pain, they demonstrated a significant reduction in pain and disability. However, it is worth noting that only two RCTs in Neto et al.’s study and three RCTs in Pourahmadi et al.’s study encompassed participants with lumbar radiculopathy, making our meta-analysis unique in its specific focus on this particular population.

### 4.1. Mechanisms of NM

Our meta-analysis uncovered that NM was more effective in reducing pain and disability when compared to control treatments. To understand how these techniques impact neural tissues, it is crucial to dive into the sequence of events that transpire when nerves encounter mechanical or chemical stimuli surpassing their tolerance threshold. Nerves possess the ability to adapt to various mechanical stresses during daily movements, but excessive stress can lead to ischemia and impaired function. Compressive stressors on nerve roots, such as disc herniation, osteophytes, or spinal stenosis, were observed to impede blood flow and cause sensory/motor dysfunction. They often result in pain due to microvascular alterations and inflammation [5]. The latter could incite nerve mechanosensitivity, which arises from inflammatory mediators and sensitizing C fibers [5]. Given the above-mentioned mechanisms of nerve injury, NM’s effectiveness can reasonably be explained in several ways.

First, NM has been investigated for its ability to induce hypoalgesia. Beneciuk et al. [9] demonstrated an immediate hypoalgesic effect on C-fiber-mediated pain following specific tensioning techniques on the median nerve, as observed in thermal quantitative sensory testing. This effect might be attributed to a decrease in glia fibrillary acid proteins in the dorsal root ganglion and lumbar spinal cord after NM, associated with reduced allodynia and hyperalgesia [47]. Second, NM has been investigated for its ability to reduce mechanosensitivity. Zhu et al. [10] reported lower concentrations of interleukin 1β (IL-1β) and tumor necrosis factor α (TNF-α) in the gluteal and trunk nerve branches following NM, correlating with reduced mechanical sensitivity. Third, there are reports showing that a promotion of nerve repair occurs after undergoing NM. In one study, increased neural growth factor and myelin protein zero levels, which play a crucial role in axonal regrowth and remyelination after injury, were demonstrated after NM [48].

### 4.2. Effectiveness of NM on Pain Reduction and Disability Improvement

NM has demonstrated benefits in reducing pain intensity in the participants analyzed in this review, whether they are in the chronic or non-chronic stage. However, when it comes to disability reduction, NM appears to be more effective in participants who are in the chronic stage. This finding can be explained by several factors. First, participants in the non-chronic stage group typically underwent shorter treatment durations (ranging from 2–3 weeks) and received fewer (i.e., three) treatment sessions per week, compared to those involved in the chronic stage group (with certain treatment durations lasting up to six weeks with up to five sessions per week). Second, nerve injuries need enough time to recover. In the early stages, nerve compression may be associated with a breakdown of the blood–nerve barrier. It could result in subperineurial edema with fibrosis and localized segmental demyelination [49]. Based on Sunderland’s classification, nerve injuries caused by mechanical stress can be categorized into multiple grades, depending on the status of continuity of the axon and myelin sheath [50]. For instance, in a first-degree injury (according to Sunderland’s classification), there is segmental demyelination, and sensory/motor functions are impaired until remyelination occurs. Full function can typically be expected without intervention within approximately 12 weeks [51]. As such, we could suppose that reparative mechanisms following nerve injury necessitate a specific recuperation period, and in the (sub)acute phases of injury, nerve repair may not have fully transpired. Lastly, because gradual damage in chronic compression does not trigger an inflammatory response, macrophages arrive slowly, often after significant Schwann cell proliferation has already occurred. This situation is associated with an increase in Schmidt–Lanterman incisures, which are cytoplasmic components of Schwann cells believed to regulate myelin sheath metabolism. Therefore, their elevated levels suggest that Schwann cells boost their metabolic activity to facilitate remyelination in response to demyelination [49]. These physiological mechanisms could have contributed to the enhanced therapeutic efficacy observed in the chronic phase.

### 4.3. Adverse Events

In the included 20 RCTs, none had reported any adverse events, which is consistent with previous systematic reviews and meta-analysis on NM [43]. However, there were some contraindications for NM, including cauda equina lesions, cord signs, and other pathologies that would affect the nervous system, such as Guillain–Barre syndrome and multiple sclerosis [52]. Therefore, it is crucial for healthcare professionals to identify these aforementioned issues before carrying out NM.

### 4.4. Limitations

It is essential to acknowledge several limitations of this study. First, we observed significant variability in the overall impact of pain and disability, indicating heterogeneity. To address this issue, subgroup analyses were conducted, focusing on different NM regimens to identify potential factors contributing to this heterogeneity. Second, our meta-analysis uncovered evidence of publication bias affecting the effect size of pain intensity and disability. To ensure the accuracy of NM’s actual effects, a subsequent meta-analysis may be warranted to confirm whether the current publication bias continues to influence NM’s true efficacy. Thirdly, lumbar radiculopathy has multifaceted origins, and among the studies included, causal factors are not uniform. Future investigations may explore the inclusion of more specific articles for a nuanced analysis. Fourthly, we acknowledge that the overall quality of the included studies is a limitation, with only seven out of the twenty studies assessed using RoB2 having a risk level lower than “high”. Future studies need to enhance the understanding of lumbar radiculopathy treatment with NM by including a greater number of high-quality RCTs. Fourthly, factors such as muscle strength, sensory impairments, or walking ability are crucial aspects of lumbar radiculopathy. Future research should further analyze and explore these factors. Lastly, due to a lack of available studies on this aspect, it is crucial to highlight that this meta-analysis could not explore whether the extent of neural pathology played role in influencing the effectiveness of NM treatment. Investigating this relationship should be considered a valuable avenue for future research exploration.

## 5. Conclusions

In summary, our analysis confirms the effectiveness of NM in reducing pain and disability in individuals with lumbar radiculopathy. Regardless of the type, NM techniques demonstrated consistent positive results for both pain reduction and disability improvement. Whether used alone or in combination with other therapies, NM was beneficial. Moreover, NM effectively reduced pain in both the chronic and non-chronic stages, with greater disability reduction observed in the chronic stage. Longer treatment duration and more frequent sessions were associated with greater improvement in pain and disability. Future studies need to focus on follow-up duration and its effects on different neural pathologies.

## Figures and Tables

**Table 1:** Characteristics of the included studies.

First Author (Year)	Country	Participants (Female/Male)	Age	Diagnosis	Duration of Symptom
Ahmed (2013) [28]	India	30	NM: 53.00 ± 1.91 Control: 52.60 ± 1.60	Sciatica	2 weeks–3 months
Anikwe (2015) [34]	Nigeria	32 (19/13)	NM: 53.50 ± 8.65 Control: 51.87 ± 10.29	Sciatica due to intervertebral disc pathology	Less than 6 weeks
Bhatia (2017) [42]	India	38	NM: 34.11 ± 8.36 Control: 35.47 ± 8.40	Lumbar radiculopathy	NM: 7.37 ± 2.85 (months) Control: 7.26 ± 2.56 (months)
Chaudhary (2022) [29]	Nepal	40 (14/26)	NM: 40.45 ± 7.3 Control: 41.5 ± 6.2	Sciatica	2 weeks–3 months
Chitra (2016) [39]	India	30 ^a^	NM: 32 ± 12.47 Control: 43.34 ± 13.12	Sciatica	NA
Čolaković (2013) [35]	Balkans	60 (33/27)	NM: 42.3 ± 6 Control: 43.1 ± 6.4	Lumbar radiculopathy	NA
Dwornik (2009) [30]	Poland	87 (52/34)	43 ± 10	Low back pain and neurogenic pain referred to the lower extremities	Chronic stage
ELDesoky (2016) [31]	Egypt	60 (22/38)	NM: 41.56 ± 4.09 Control: 40.8 ± 5.37	Herniated or bulged disc, or foraminal stenosis at L5-S1 level were the causes of radiculopathy	More than 3 months
Ferreira (2016) [36]	Brazil	60 (45/15) ^a^	NM: 43.9 ± 14.5 Control: 40.3 ± 12.9	Unilateral nerve-related leg pain	At least 12 weeks
Geethika (2015) [37]	India	30	30–50	Pain or paresthesia in lumbar spine with radiating pain to lower extremity	Sub-acute or chronic stage
Jeong (2016) [40]	Korea	30 (14/16)	NM: 35.1 ± 6.4 Control: 41.6 ± 11.1	Low back pain patients with radiating lower limb pain	NA
Morsi (2022) [27]	Egypt	24 (22/14)	NM: 34.38 ± 7.25 Control: 34.92 ± 6.46	Discogenic sciatica	More than 12 weeks up to 1 year
Pallipamula (2012) [43]	India	42 ^a^	NM: 42.53 ± 6.99 Control: 40.2 ± 7.55	Sciatica	NM: 63.63 ± 13.20 (days) Control: 62.4 ± 12.58 (days)
Plaza-Manzano (2020) [41]	Spain	32 (16/16)	NM: 47.0 ± 8.0 Control: 45.5 ± 6.0	Disc herniation between L4 and S1 levels with lumbar radiating pain to one lower limb including the foot	NM: 17.2 ± 1.5 (months) Control: 17.3 ± 1.4 (months)
Sarkari (2007) [44]	India	30 (16/14)	NM: 56.1 ± 4.95 Control: 58.3 ± 4.37	Sciatica	NA
Sharma (2018) [32]	India	21 (13/11)	NM: 38.50 ± 5.73 Control: 37.55 ± 7.59	Lumbosacral radiculopathy	NM: 3.5 + 1.00 (months) Control: 4.0 + 1.00 (months)
Tella (2022) [46]	Nigeria	32 (19/13)	NM: 53.50 ± 8.65 Control: 51.87 ± 10.29	Sciatica due to intervertebral disc pathology	Acute stage
Vijayalakshmi (2022) [38]	India	23 (15/8)	NM: 41.1 ± 8.3 Control: 40.2 ± 6.2	Low back pain with radiating pain distal to leg	Less than 3 months
Zahid (2014) [45]	Pakistan	94	20–60	Sciatica	2 weeks–3 months
Zainab (2022) [33]	Pakistan	80 ^a^	NM: 39.42 ± 7.62 Control: 38.13 ± 8.03	Lumbosacral radiculopathy	More than 2 months

Age and pain duration are presented as mean ± standard deviation or range; NA, not available; NM, neural mobilization; ^a^ allocated participants.

**Table 2:** Summary of the intervention details of the included trials.

First Author, Year	NM Group (Per-Protocol N)	Control Group (Per-Protocol N)	NM Treatment Protocol	Outcome Measurement
Ahmed (2013) [28]	NM + conventional treatment (15)	Conventional treatments (15) (Lumbar extension/flexion exercise plus TENS)	Total: 2 weeks, 3 days/week NM: SLR technique, slider, 2 sets of 20 repetitions	NRS SF-12
Anikwe (2015) [34]	NM + physical agents + massage (16)	Physical agents + massage (16)	Total intervention period: 2 weeks, 3 days/week NM: slump technique, slider, 15 times for 3 sets with an interval of 5 min between each set	NRS
Bhatia (2017) [42]	NM + lumbar stabilization exercise (19)	Lumbar stabilization exercise (19)	Total: 4 weeks, 5 days/week NM: slump technique, slider, 5 sets of 15 repetitions	NRS RMDQ
Chaudhary (2022) [29]	NM + conventional treatment (20)	Conventional treatments (20) (Physical agents, piriformis stretch, lumbar extension exercise)	Total: 4 weeks, 3 days/week NM: SLR technique, slider, repetitions not mentioned	VAS ODI
Chitra (2016) [39]	NM + TENS (14)	Kinesio taping (14)	Total: 2 weeks, 3 days/week NM: technique not mentioned, grade 4 Maitland mobilization for all branches of sciatic nerve, repetitions not mentioned	VAS
Čolaković (2013) [35]	NM + lumbar stabilization exercise (30)	Active ROM exercise + lumbar stabilization exercises (30)	Total intervention period: 4 weeks, 3 days/week NM: side-lying SLR technique, slider, repeated 3 times with 10 oscillatory movements	VAS
Dwornik (2009) [30]	NM only (42)	Conventional treatments (45) (Physical agent and lumbar exercise)	Total: 2 weeks NM: tensioner and mobilization techniques, repetitions not mentioned	VAS
ELDesoky, 2016 [31]	NM + conventional treatments (30)	Conventional treatments (30) (physical agents, lumbar extension)	Total: 6 weeks, 3 days/week NM: SLR technique, including 30 s oscillations and 1 min rest in each session	VAS ODI
Ferreira (2016) [36]	NM + lumbar mobilization (28)	Education about ADL (28)	Total intervention period: 2 weeks, 2 days/week NM: side-lying SLR and slump, slider, two sets of 30 repetitions	VAS ODI
Geethika (2015) [37]	NM + conventional treatments + hamstring stretching + trigger release (15)	Conventional treatments + hamstring stretching + trigger release (lumbar traction + cryotherapy + back-strengthening exercises) (15)	Total intervention period: 3 weeks, 3 days/week NM: SLR technique, 10 min per session including 30 s hold and 1 min rest	VAS ODI
Jeong (2016) [40]	NM + lumbar segmental stabilization exercise (15)	Lumbar segmental stabilization exercise (15)	Total intervention period: 6 weeks, 3 days/week NM: technique and number of repetitions not mentioned	SF-36
Morsi (2022) [27]	NM only (24)	Stretching lower extremity muscle (12)	Total: 2 weeks, 3 days per week NM: slump technique, slider and tensioner, 3 sets in every session	VAS ODI
Pallipamula (2012) [43]	NM + physical agents (19)	Physical agents (20)	Total intervention period: 6 days, once daily NM: slump technique, slider, participant performs knee extension with neck extension with hold for 5 s and then flexes both the knee and neck simultaneously and holds it for 5 s	VAS MODI
Plaza-Manzano (2020) [41]	NM+ lumbar stabilization exercise (16)	Lumbar stabilization exercise (16)	Total intervention period: 4 weeks, 2 days/week NM: SLR technique, slider, 3 sets of 10 repetitions in each treatment session	NRS RMDQ
Sarkari (2007) [44]	NM + physical agents (15)	Physical agents (15)	Total: 9 sessions NM: SLR, 10 min per session including 30 s hold and 1 min rest	VAS
Sharma (2018) [32]	NM + conventional treatments (11)	Conventional treatment (10) (Hot back, lumbar strengthening)	Total: 6 sessions NM: slider and tensioner techniques, number of repetitions not mentioned	NRS MODI
Tella (2022) [46]	NM+ conventional treatment + massage (16)	Conventional treatment + massage (16) (TENS + lumbar extension exercise)	Total: 2 weeks, 3 days/week NM: slump technique, slider, 15 times for 3 sets with an interval of 5 min	NRS
Vijayalakshmi (2022) [38]	NM + conventional treatments + hamstring stretching (13)	Conventional treatments + hamstring stretching (10) (interferential therapy lumbar strengthening)	Total: 3 weeks, total 10 sessions NM: slump technique, both sliders and tensioners, nerve sliding technique was applied for 20–30 repetitions in 2–3 sets per day for 10 sessions, and nerve tensioning technique was also implemented for 15–25 s in 5–7 repetitions in sessions 8–10.	NRS ODI
Zahid (2014) [45]	NM + physical agents (47)	Physical agents (47)	Total: 9 sessions NM: SLR technique, neural mobilization was given for 10 min/session, including 30 s hold and 1 min rest	NRS QBPDS
Zainab (2022) [33]	NM + conventional treatments (40)	Conventional treatments (37) (Physical agents, lumbar strengthening)	Total: 2 weeks, 3 days/week NM: SLR technique, 3 sets of 10 oscillatory movements	NRSMODI

MODI, Modified Oswestry Disability Index; NM, neural mobilization; NRS, Numeric Rating Scale; ODI, Oswestry Disability Index; QBPDS, Quebec Back Pain Disability Scale; RMDQ, Roland Morris Disability Questionnaire; SF-36, 36-Item Short Form Survey; SF-12, 12-Item Short Form Survey; TENS, Transcutaneous Electrical Nerve Stimulation; VAS, Visual Analog Scale.

**Table 3:** Detailed quality assessment of included studies using Cochrane risk-of-bias 2 tool.

First Author	Year	Randomization Process	Intervention Adherence	Missing Outcome Data	Outcome Measurement	Selective Reporting	Overall RoB
Ahmed	2013	H ^1,4^	L	H ^5^	L	L	H
Anikwe	2015	L	L	L	L	L	L
Bhatia	2017	S ^4^	L	H ^5^	L	L	H
Chaudhary	2022	S ^4^	L	H ^5^	L	L	H
Chitra	2016	S ^4^	L	S ^3^	L	L	H
Čolaković	2013	S ^1^	L	H ^5^	L	L	H
Dwornik	2009	H ^1,4^	L	H ^5^	L	L	H
ELDesoky	2016	L	L	H ^5^	L	L	H
Ferreira	2016	S ^4^	L	S ^2^	L	L	H
Geethika	2015	H ^1,4^	L	H ^5^	L	L	H
Jeong	2016	S ^1^	L	H ^5^	L	L	H
Morsi	2022	L	L	H ^5^	L	L	H
Pallipamula	2012	L	L	S ^6^	L	L	S
Plaza-Manzano	2020	L	L	L	L	L	L
Sarkari	2007	S ^1^	L	L	L	L	S
Sharma	2018	S ^1^	L	S ^6^	L	L	H
Tella	2022	L	L	S ^3^	L	L	S
Vijayalakshmil	2022	L	L	L	L	L	L
Zahid	2014	S ^4^	L	H ^5^	L	L	H
Zainab	2022	L	L	S ^6^	L	L	S

^1^ There was no proper allocation concealment reported; ^2^ six subjects discontinued intervention and missed assessment; ^3^ two subjects discontinued intervention and missed assessment; ^4^ there were no significant or insignificant differences in demographics or baseline characteristics of the participants reported; ^5^ there was no information about the extent of missing outcome data; ^6^ three subjects discontinued intervention and missed assessment; H: high risk; S: some risk; L: low risk.

## Data Availability

The datasets used and/or analyzed during the current study are available from the corresponding author on reasonable request.

## Supplementary Materials

The following supporting information can be downloaded at: https://www.mdpi.com/article/10.3390/life13122255/s1. Table S1: PRISMA checklist; Table S2: keywords and search results in different databases; Table S3: description of primary outcome measurements; Table S4: excluded studies and reasons; Figure S1: summary of quality assessment; Figure S2: sensitivity analysis for pain reduction; Figure S3: meta-regression analysis of treatment duration for pan reduction; Figure S4: meta-regression analysis of sessions per week for pan reduction; Figure S5: sensitivity analysis for disability improvement; Figure S6: meta-regression analysis of treatment duration for disability improvement; Figure S7: meta-regression analysis of sessions per week for disability improvement; Figure S8: funnel plot of pain reduction; Figure S9: funnel plot of disability improvement.
