# Supplementary material for: Neural Mobilization for Reducing Pain and Disability in Patients with Lumbar Radiculopathy: A Systematic Review and Meta-Analysis
Source: Life (Basel). 2023 Nov 26;13(12):2255. doi: 10.3390/life13122255 (PMC10744707; doi:10.3390/life13122255)

Supplemental Material

Table S1. PRISMA Checklist

Section and Topic	#	Checklist item	Location
		**TITLE**	
Title	1	Identify the report as a systematic review.	Title
		**ABSTRACT**	
Abstract	2	See the PRISMA 2020 for Abstracts checklist.	Abstract
		**INTRODUCTION**	
Rationale	3	Describe the rationale for the review in the context of existing knowledge.	Introduction
Objectives	4	Provide an explicit statement of the objective(s) or question(s) the review addresses.	Introduction
		**METHODS**	
Eligibility criteria	5	Specify the inclusion and exclusion criteria for the review and how studies were grouped for the syntheses.	Methods
Information sources	6	Specify all databases, registers, websites, organisations, reference lists and other sources searched or consulted to identify studies. Specify the date when each source was last searched or consulted.	Methods
Search strategy	7	Present the full search strategies for all databases, registers and websites, including any filters and limits used.	Methods, Table S2
Selection process	8	Specify the methods used to decide whether a study met the inclusion criteria of the review, including how many reviewers screened each record and each report retrieved, whether they worked independently, and if applicable, details of automation tools used in the process.	Methods
Data collection process	9	Specify the methods used to collect data from reports, including how many reviewers collected data from each report, whether they worked independently, any processes for obtaining or confirming data from study investigators, and if applicable, details of automation tools used in the process.	Methods
Data items	10a	List and define all outcomes for which data were sought. Specify whether all results that were compatible with each outcome domain in each study were sought (e.g., for all measures, time points, analyses), and if not, the methods used to decide which results to collect.	Methods

	10b	List and define all other variables for which data were sought (e.g., participant and intervention characteristics, funding sources). Describe any assumptions made about any missing or unclear information.	Methods Table 1
Study risk of bias assessment	11	Specify the methods used to assess risk of bias in the included studies, including details of the tool(s) used, how many reviewers assessed each study and whether they worked independently, and if applicable, details of automation tools used in the process.	Methods
Effect measures	12	Specify for each outcome the effect measure(s) (e.g., risk ratio, mean difference) used in the synthesis or presentation of results.	Methods
Synthesis methods	13a	Describe the processes used to decide which studies were eligible for each synthesis (e.g., tabulating the study intervention characteristics and comparing against the planned groups for each synthesis (item #5)).	Methods, Figure 1, Table 1, Table S4
	13b	Describe any methods required to prepare the data for presentation or synthesis, such as handling of missing summary statistics, or data conversions.	Methods
	13c	Describe any methods used to tabulate or visually display results of individual studies and syntheses.	Methods
	13d	Describe any methods used to synthesize results and provide a rationale for the choice(s). If meta-analysis was performed, describe the model(s), method(s) to identify the presence and extent of statistical heterogeneity, and software package(s) used.	Methods
	13e	Describe any methods used to explore possible causes of heterogeneity among study results (e.g., subgroup analysis, meta-regression).	Methods
	13f	Describe any sensitivity analyses conducted to assess robustness of the synthesized results.	Methods
Reporting bias assessment	14	Describe any methods used to assess risk of bias due to missing results in a synthesis (arising from reporting biases).	Methods, Figure S1, Table 3
Certainty assessment	15	Describe any methods used to assess certainty (or confidence) in the body of evidence for an outcome.	Methods
**RESULTS**			
Study selection	16a	Describe the results of the search and selection process, from the number of records identified in the search to the number of studies included in the review, ideally using a flow diagram.	Results, Figure 1, Table S2-S4

	16b	Cite studies that might appear to meet the inclusion criteria, but which were excluded, and explain why they were excluded.	Results, Table S4
Study characteristics	17	Cite each included study and present its characteristics.	Results, Table 1
Risk of bias	18	Present assessments of risk of bias for each included study.	Figure S1, Table 2
Results of individual studies	19	For all outcomes, present, for each study: (a) summary statistics for each group (where appropriate) and (b) an effect estimates and its precision (e.g., confidence/credible interval), ideally using structured tables or plots.	Figure 2-5, Figure S2-S9
Results of syntheses	20a	For each synthesis, briefly summarise the characteristics and risk of bias among contributing studies.	Results, Table 3
	20b	Present results of all statistical syntheses conducted. If meta-analysis was done, present for each the summary estimate and its precision (e.g., confidence/credible interval) and measures of statistical heterogeneity. If comparing groups, describe the direction of the effect.	Results, Figure 2-5, Figure S2-S9
	20c	Present results of all investigations of possible causes of heterogeneity among study results.	Results, Figure 2-5, Figure S2-S9
	20d	Present results of all sensitivity analyses conducted to assess the robustness of the synthesized results.	Results, Figure S2 Figure S5
Reporting biases	21	Present assessments of risk of bias due to missing results (arising from reporting biases) for each synthesis assessed.	Figure S1, Table 3
Certainty of evidence	22	Present assessments of certainty (or confidence) in the body of evidence for each outcome assessed.	Figure 2-5, Figure S2-S9

**DISCUSSION**			
Discussion	23a	Provide a general interpretation of the results in the context of other evidence.	Discussion
	23b	Discuss any limitations of the evidence included in the review.	Discussion
	23c	Discuss any limitations of the review processes used.	Discussion
	23d	Discuss implications of the results for practice, policy, and future research.	Discussion
**OTHER INFORMATION**			
Registration and protocol	24a	Provide registration information for the review, including register name and registration number, or state that the review was not registered.	Methods
	24b	Indicate where the review protocol can be accessed, or state that a protocol was not prepared.	Methods, Table S2-S4
	24c	Describe and explain any amendments to information provided at registration or in the protocol.	Methods, Table S2-S4
Support	25	Describe sources of financial or non-financial support for the review, and the role of the funders or sponsors in the review.	Funding
Competing interests	26	Declare any competing interests of review authors.	Conflicts of interest
Availability of data, code and other materials	27	Report which of the following are publicly available and where they can be found: template data collection forms; data extracted from included studies; data used for all analyses; analytic code; any other materials used in the review.	Results, Table S2-S4

Table S2. Keywords and search results in different databases

Database	Keyword	Filter	Date	Results
PubMed	: ("neural mobilization techniques" OR "neurodynamic mobilization techniques" OR "nerve mobilization techniques") AND ("lumbar radiculopathy" OR "sciatica")	Clinical trial	October 6, 2023	458
Cochrane Library	: ("neural mobilization techniques" OR "neurodynamic mobilization techniques" OR "nerve mobilization techniques") AND ("lumbar radiculopathy" OR "sciatica")	Title Abstract Keyword	October 6, 2023	1423
Clinical Trials .gov	: ("neural mobilization techniques" OR "neurodynamic mobilization techniques" OR "nerve mobilization techniques") AND ("lumbar radiculopathy" OR "sciatica")	Condition or disease	October 6, 2023	24
PEDro	: ("neural mobilization techniques" OR "neurodynamic mobilization techniques" OR "nerve mobilization techniques") AND ("lumbar radiculopathy" OR "sciatica")	Condition or disease	October 6, 2023	3

Table S3. Detail description of primary outcome measurements and secondary outcome measurements

Primary outcome measurements	Description
Numeric Rating Scale (NRS)	Included options with either 11 points (NRS-11) or 101 points (NRS-101) were used to assess the level of pain.
Visual Analog Scale (VAS)	10-centimeter line ranging from 'no pain' to 'worst possible pain' to capture pain intensity.
**Secondary outcome measurements**	**Description**
Oswestry Disability Index (ODI)	● 10 everyday activities.<br>● Each activity has six statements scored from 0 (least disability) to 5 (greatest disability).<br>● The total score is a percentage: 0% means no disability, while 100% means the highest level of disability
Modified Oswestry Disability Index (MODI)	● Incorporating two additional inquiries concerning forward bending.<br>● Incorporating two additional inquiries concerning occupational status.<br>● Excluding questions pertaining to sexual activity, weight lifting, or travel in ODI.
Quebec Back Pain Disability Scale (QBPDS)	● 20 daily activities across six categories.<br>● Each activity is rated on a scale of 0-5 (0 = no effort, 5 = unable to).<br>● Scores range from 0 to 100, reflecting the level of functional disability.<br>● Higher scores indicate more disability.
Roload Morris Disability Questionnaire (RMDQ)	Assesses 24 daily activities and the score ranges from 0 (no disability) to 24 (max. disability)
36-Item Short Form Survey (SF-36)	● 36 questions covering eight domains of health.<br>● A higher score corresponds to less disability.<br>● For example, a score of 0 is equivalent to maximum disability, while a score of 100 is equivalent to no disability.
12- Item Short Form Survey (SF-12)	Contains 12 items rather than 36

Table S4. Excluded studies and reasons

Citations	Reasons
Alshami, A. M., Alghamdi, M. A., & Abdelsalam, M. S. (2021). Effect of Neural Mobilization Exercises in Patients With Low Back-Related Leg Pain With Peripheral Nerve Sensitization: A Prospective, Controlled Trial. Journal of chiropractic medicine, 20(2), 59–69. https://doi.org/10.1016/j.jcm.2021.07.001	Not a randomized controlled trial
Santos De Melo, L., & Nogueira, L. A. C. (2019). Pragmatic neural tissue management improves short-term pain and disability in patients with sciatica: a single-arm clinical trial. The Journal of manual & manipulative therapy, 27(4), 208–214. https://doi.org/10.1080/10669817.2019.1580420	Not a randomized controlled trial
Silva, L. I., Rocha, B. P., Antunes, J. S., Karvat, J., Kakihata, C. M. M., Mattjie, T. F., & Bertolini, G. R. F. (2013). Evaluation of the pressure pain threshold after neural mobilization in individuals with sciatica. The European Journal of Physiotherapy, 15(3), 146-150.	Not a randomized controlled trial
Karvat, J., Antunes, J. S., Bernardino, G. R., Kakihata, C. M. M., & Bertolini, G. R. F. (2014). Effect of low-level LASER and neural mobilization on nociceptive threshold in experimental sciatica. Revista Dor, 15, 207-210.	Not a randomized controlled trial
Bertolini, G. R., Silva, T. S., Trindade, D. L., Ciena, A. P., & Carvalho, A. R. (2009). Neural mobilization and static stretching in an experimental sciatica model: an experimental study. Brazilian Journal of Physical Therapy, 13, 493-498.	Not a randomized controlled trial
Shaker, H., & Abd El-Mageed, S. (2008). Effect of neurodynamic mobilization on chronic discogenic sciatica. Bull. Fac. Ph. Th. Cairo Univ, 13(1), 153-161.	Not a randomized controlled trial
Gupta, M. (2012). Effectiveness of nerve mobilization in the management of sciatica. Physiotherapy and Occupational Therapy, 6(2), 79.	Not available data of pre- and post- intervention pain/disability assessment nor change pain/disability score
K, Kotteeswaran & Virupakshi, G.. (2017). Efficacy of neurodynamic treatment on pain and rom (SLR) in subjects with low back pain associated with sciatica. Biomedicine (India). 37. 382-387.	Not available data of pre- and post- intervention pain/disability assessment nor change pain/disability score (Despite attempting to obtain the full text through national libraries, internet searches, and contacting the author, it remains unavailable)

Ibrahiem, B. M., Labib, A. M., Nasef, S. A. S., & Said, S. M. A. (2017). Impact of different neurodynamic tension techniques on H reflex of sciatic nerve. Journal of Medical Sciences (Faisalabad), 17(2), 68-74.	Did not report pain intensity and disability
Danazumi, M. S., Nuhu, J. M., Ibrahim, S. U., Falke, M. A., Rufai, S. A., Abdu, U. G., Adamu, I. A., Usman, M. H., Daniel Frederic, A., & Yakasai, A. M. (2023). Effects of spinal manipulation or mobilization as an adjunct to neurodynamic mobilization for lumbar disc herniation with radiculopathy: a randomized clinical trial. The Journal of manual & manipulative therapy, 1–13. Advance online publication. https://doi.org/10.1080/10669817.2023.2192975	Lacking a control group not using neural mobilization
Ismail, M. M., Ayad, K. E., Sharaf, M. A., & Hakeem, M. G. (2009). Low Energy Laser Therapy and Nerve Mobilization in Sciatica. Bull. Fac. Ph. Th. Cairo Univ, 14(1), 35.	Lacking a control group not using muscle neural mobilization
Rehman, A., Afzal, B., Hassan, D., Malik, A. N., & Noor, R. (2022). Effects of Active and Passive Lower Extremity Neural Mobilization on Pain and Functional Level in Patients with Lumber Radiculopathy. Pakistan Journal of Medical Research, 61(1), 19-23.	Lacking a control group not using muscle neural mobilization
Bhatt, K., & Shukla, Y. Effects of Two Neural Mobilization Techniques in Sciatica: A Comparative Study.	Lacking a control group not using muscle neural mobilization
Salam, A., Khalid, A., Waseem, I., Mahmood, T., & Mahmood, W. (2022). Comparison between effects of passive versus self-mobilization of sciatic nerve in piriformis syndrome for relieving pain and improving hip outcomes.: soi: 21-2017/re-trjvol06iss01p298. The Rehabilitation Journal, 6(01), 298-302.	Lacking a control group not using muscle neural mobilization
Cleland, J. A., Childs, J. D., Palmer, J. A., & Eberhart, S. (2006). Slump stretching in the management of non-radicular low back pain: a pilot clinical trial. Manual therapy, 11(4), 279–286. https://doi.org/10.1016/j.math.2005.07.002	Inclusion criteria did not consisted in lumbar radiculopathy or sciatica specifically
Nagrale, A. V., Patil, S. P., Gandhi, R. A., & Learman, K. (2012). Effect of slump stretching versus lumbar mobilization with exercise in subjects with non-radicular low back pain: a randomized clinical trial. The Journal of manual & manipulative therapy, 20(1), 35–42. https://doi.org/10.1179/2042618611Y.0000000015	Inclusion criteria did not consist lumbar radiculopathy or sciatica specifically
BASSEM, G. E. N., HAYTHAM, I. M., & Ibrahim, M. (2021). Difference between Neurodynamic Mobilization and Stretching Exercises for Chronic Discogenic Sciatica. The Medical Journal of Cairo University, 89(September), 1869-1876.	Participants overlapped with another publication of the author (Osama 2020)

Figure S1. Summary of quality assessment of studies included in the meta-analysis using Cochrane risk of bias 2 tool

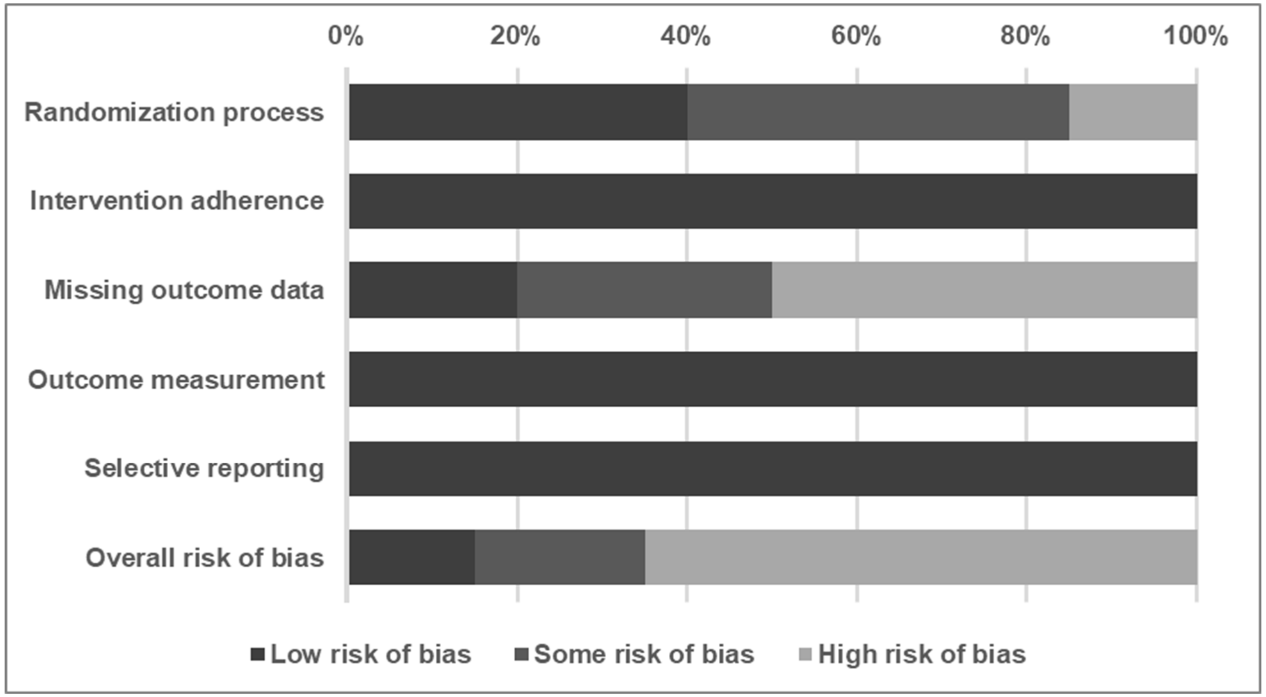

Figure S2. Results of sensitivity analysis using the one-study removal method to assess the impact of neural mobilization (NM) on the overall effect size for pain reduction

Study name	Statistics with study removed			
	Point	Lower limit	Upper limit	p-Value
Ahmed 2013	-1.072	-1.468	-0.676	0.000
Anikwe 2015	-1.055	-1.445	-0.664	0.000
Bhatia 2017	-1.124	-1.531	-0.718	0.000
Chaudhary 2022	-1.086	-1.487	-0.684	0.000
Chitra 2016	-1.114	-1.518	-0.711	0.000
Colakovic 2013	-1.138	-1.548	-0.728	0.000
Dwornik, 2009	-1.133	-1.550	-0.715	0.000
ELDesoky 2016	-1.019	-1.388	-0.651	0.000
Ferreira 2016	-1.144	-1.551	-0.737	0.000
Geethika 2015	-1.094	-1.496	-0.693	0.000
Morsi 2022	-1.084	-1.484	-0.684	0.000
Pallipamula 2012	-1.055	-1.445	-0.664	0.000
Plaza-Manzano 2020	-1.100	-1.503	-0.697	0.000
Sarkari 2007	-1.007	-1.377	-0.637	0.000
Sharma, 2018	-1.105	-1.505	-0.704	0.000
Tella 2022	-1.055	-1.445	-0.664	0.000
Vijayalakshmi 2022	-1.112	-1.513	-0.710	0.000
Zahid 2014	-1.182	-1.527	-0.838	0.000
Zainab 2022	-1.163	-1.559	-0.768	0.000
	-1.097	-1.482	-0.712	0.000

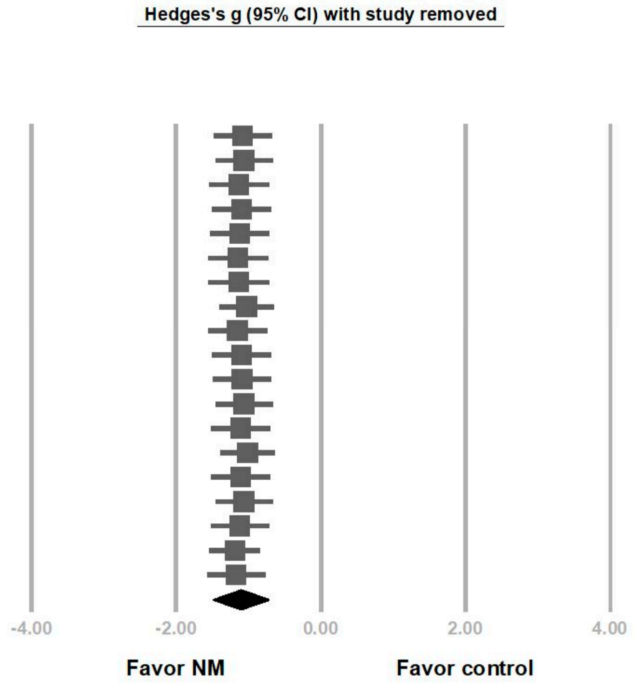

Figure S3. Meta-regression analysis showing the relationship between the duration of neural mobilization (NM) in days and the magnitude of pain reduction

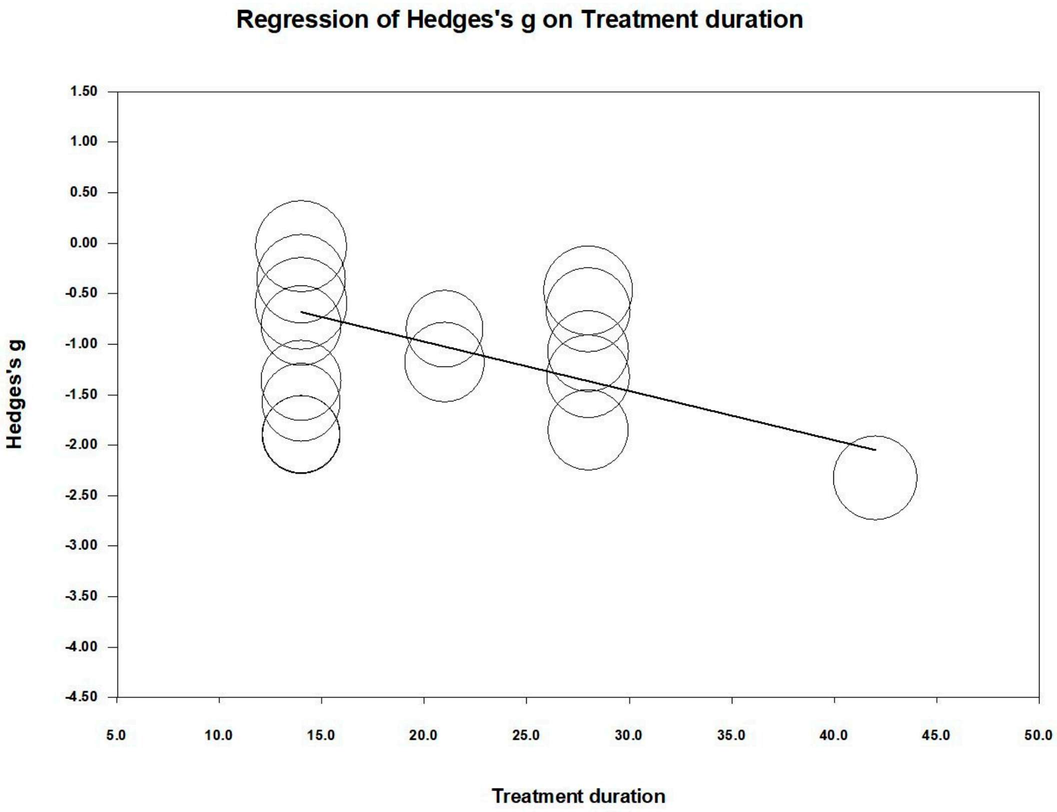

Figure S4. Meta-regression analysis showing the relationship between the sessions per week of neural mobilization (NM) and the magnitude of pain reduction

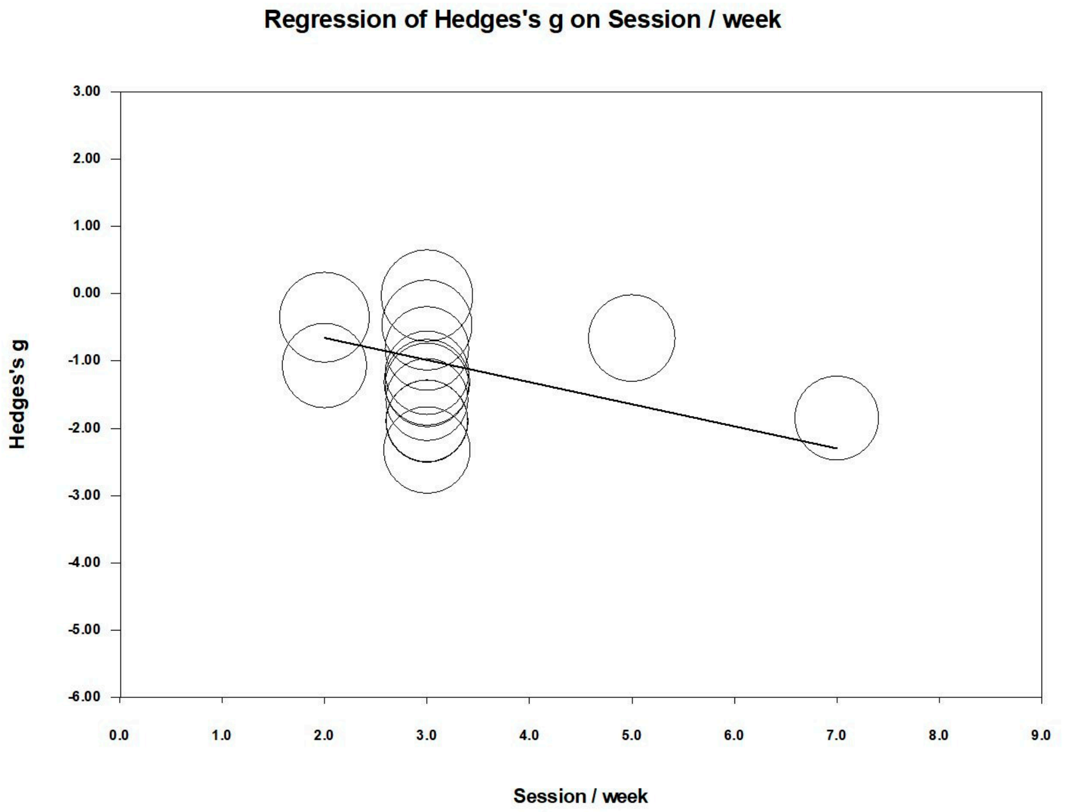

Figure S5. Results of sensitivity analysis using the one-study removal method to assess the impact of neural mobilization (NM) on the overall effect size for relief of disability

Study name	Statistics with study removed			
	Point	Lower limit	Upper limit	p-Value
Ahmed 2013	-0.954	-1.497	-0.411	0.001
Bhatia 2017	-0.989	-1.542	-0.437	0.000
Chaudhary 2022	-0.944	-1.488	-0.401	0.001
ELDesoky 2016	-0.825	-1.287	-0.364	0.000
Ferreira 2016	-1.013	-1.572	-0.453	0.000
Geethika 2015	-0.957	-1.500	-0.414	0.001
Jeong 2016	-0.976	-1.522	-0.429	0.000
Morsi 2022	-0.891	-1.410	-0.372	0.001
Pallipamula 2012	-0.867	-1.371	-0.362	0.001
Plaza-Manzano 2020	-0.955	-1.499	-0.411	0.001
Sharma, 2018	-0.998	-1.540	-0.456	0.000
Vijayalakshmi 2022	-0.979	-1.522	-0.436	0.000
Zahid, 2014	-1.089	-1.548	-0.630	0.000
Zainab 2022	-1.058	-1.592	-0.524	0.000
	-0.964	-1.475	-0.453	0.000

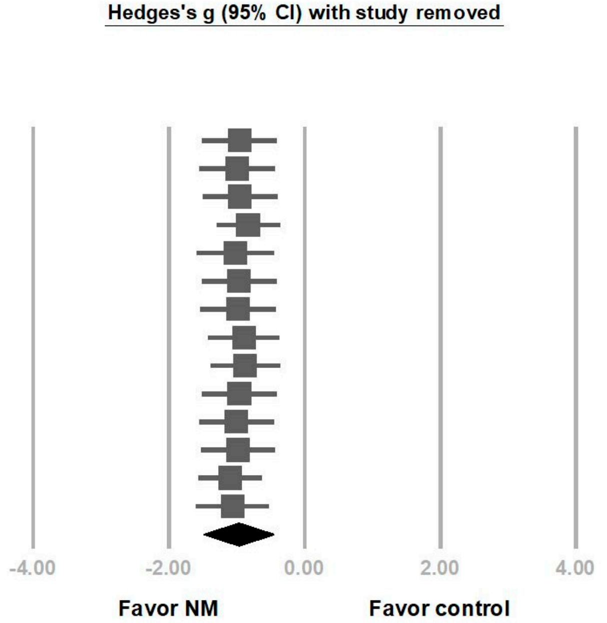

Figure S6. Meta-regression analysis showing the relationship between the duration of neural mobilization (NM) in days and the magnitude of disability improvement

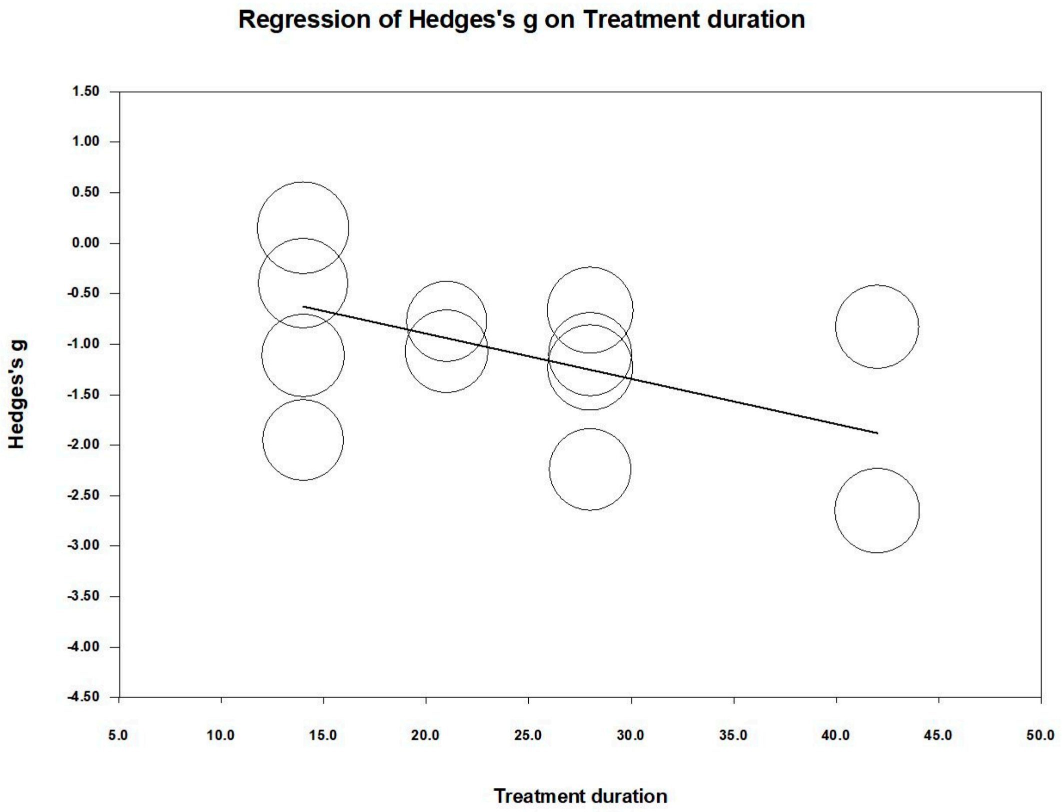

Figure S7. Meta-regression analysis showing the relationship between the sessions per week of neural mobilization (NM) and the magnitude of disability improvement

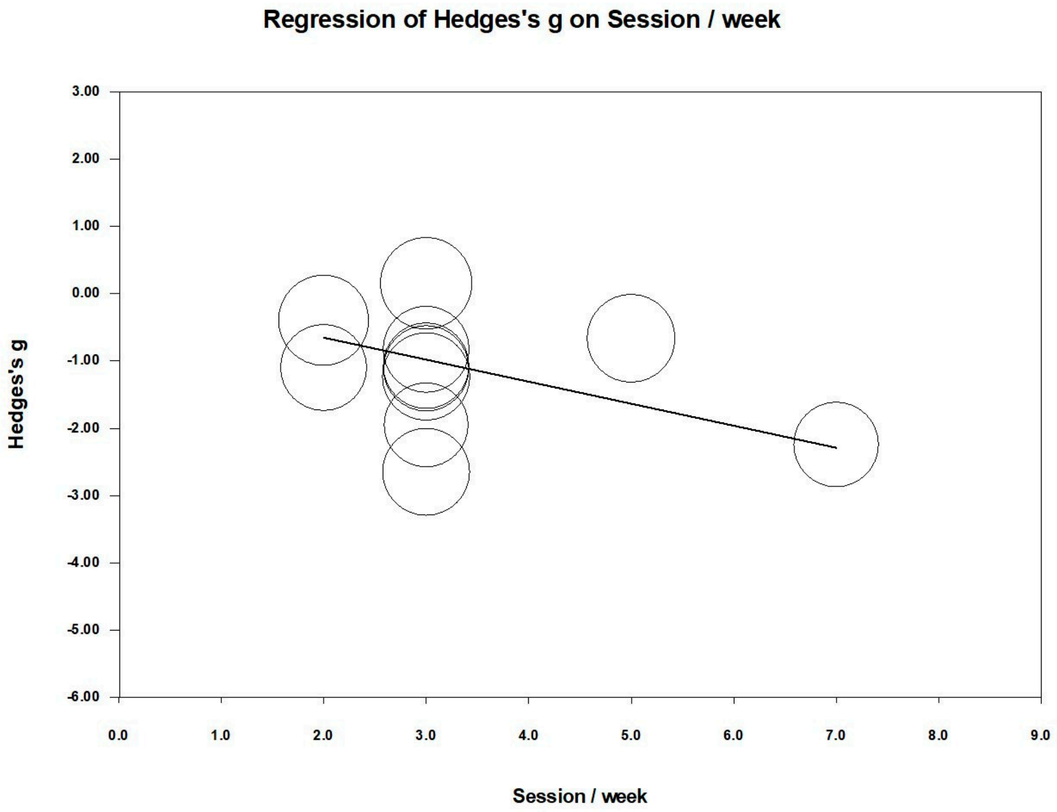

Figure S8. Funnel plot depicting the distribution of effect sizes for pain reduction across studies

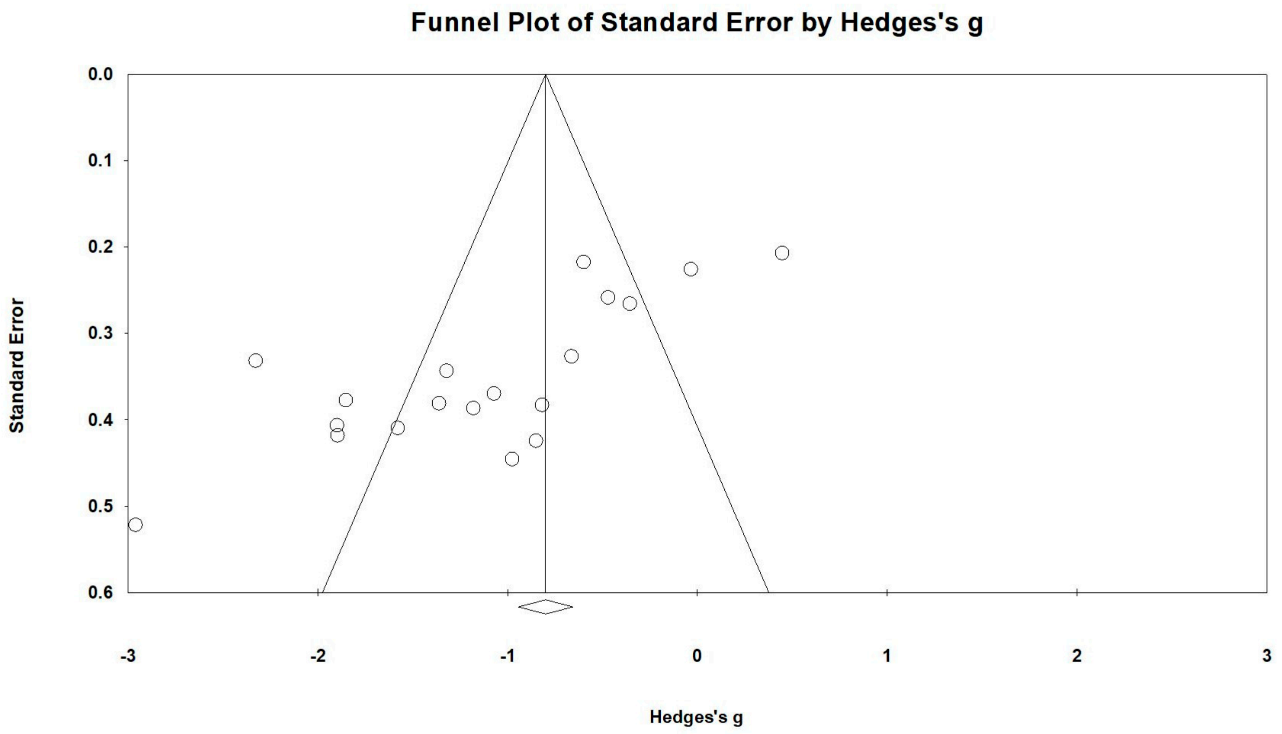

Figure S9. Funnel plot depicting the distribution of effect sizes for reduction of disability across studies

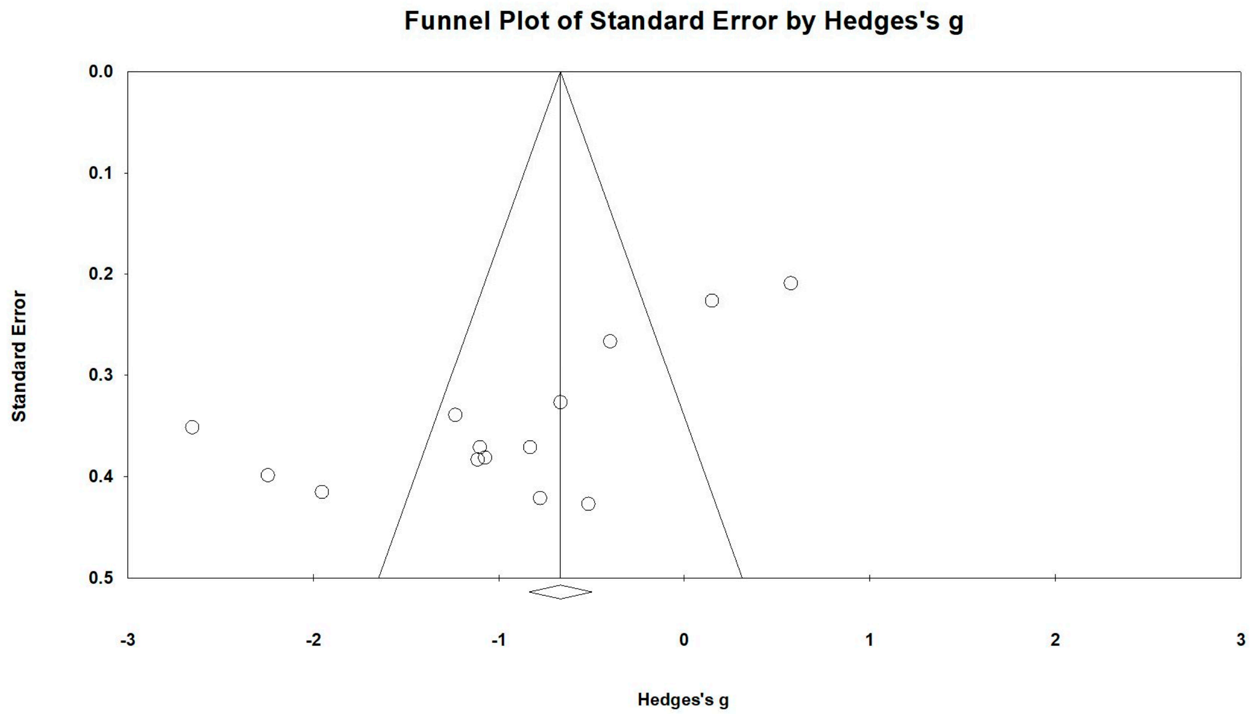

Supplement: Supplementary file 1 [file life-13-02255-s001.zip › life-2701319-supplementary.pdf]
